# Expert consensus on fetal ventriculomegaly: evidence-based recommendations for 23 key clinical questions

**DOI:** 10.3389/fped.2025.1678359

**Published:** 2025-10-17

**Authors:** Xin Li, Ting Hu, Xue Xiao

**Affiliations:** ^1^ Department of Medical Genetics, West China Second University Hospital, Sichuan University, Chengdu, Sichuan, China; ^2^ Key Laboratory of Birth Defects and Related Diseases of Women and Children (Sichuan University), Ministry of Education, West China Second Hospital, Sichuan University, Chengdu, China; ^3^ Department of Obstetrics, West China Second University Hospital, Sichuan University, Chengdu, Sichuan, China

**Keywords:** fetal ventriculomegaly, prenatal diagnosis, Delphi, chromosomal microarray, expert consensus

## Abstract

**Background:**

Fetal ventriculomegaly (VM), defined as an atrial diameter ≥10 mm, is one of the most frequently identified central nervous system anomalies on prenatal imaging. This expert consensus aims to address current gaps and inconsistencies in the prenatal diagnosis and management of fetal VM by providing evidence-based, graded recommendations across five key domains: diagnosis and etiology, systematic evaluation, antenatal management, delivery considerations, and short- and long-term prognosis.

**Methods:**

A multidisciplinary panel employed a modified Delphi method to formulate and refine 23 critical clinical questions. The process involved iterative rounds of expert consultation, structured questionnaires, and consensus building among specialists in obstetrics, fetal imaging, genetics, neonatology, neurology, rehabilitation, nursing, and informatics. Recommendations were informed by current international guidelines, high-quality cohort studies, and meta-analyses, and were graded using a modified GRADE framework to reflect the strength and quality of supporting evidence.

**Results:**

Key recommendations include the standardized use of ultrasound and fetal MRI, the application of chromosomal microarray (CMA) in all VM cases regardless of isolation status, individualized monitoring protocols based on ventricular progression, and the need for structured neurodevelopmental follow-up in selected high-risk cases. Novel insights highlight the potential role of dynamic imaging parameters, maternal systemic factors, and emerging multi-omics tools in risk stratification and etiological investigation.

**Conclusion:**

This consensus provides a comprehensive, structured approach to fetal VM, promoting standardized clinical practice and facilitating early identification of high-risk fetuses. It emphasizes multidisciplinary decision-making and calls for future research into prognostic scoring systems, long-term outcomes, and novel etiological pathways.

## Background

Fetal ventriculomegaly is one of the most frequently identified abnormalities in prenatal imaging, particularly during the second and third trimesters. Although many cases—especially those with mild to moderate dilation—have favorable outcomes, uncertainties remain regarding optimal diagnostic thresholds, etiologic classification, and risk stratification (1). Borderline measurements (e.g., 12–13 mm), asymmetric or progressive dilation, and the influence of maternal factors complicate clinical decision-making (2). At the same time, advances in fetal magnetic resonance imaging (MRI) and genomic technologies have expanded the tools available for evaluating underlying causes, yet their application varies widely across clinical settings (3, 4).

A consensus has yet to be established regarding the optimal management of pregnancies affected by isolated or complex ventriculomegaly, particularly concerning the timing and mode of delivery, antenatal surveillance protocols, and the necessity for neonatal and long-term neurodevelopmental follow-up (5). To address these gaps, this expert consensus document was developed through a structured Delphi process, engaging a multidisciplinary panel of experts in maternal-fetal medicine, radiology, genetics, neonatology, and pediatric neurology. Unlike existing guidelines that provide overarching principles, this consensus adopts a structured, question-based approach to offer practical, graded recommendations for specific clinical scenarios, thereby enhancing usability in routine care. The consensus is organized into five key domains—diagnosis, evaluation, antenatal management, delivery, and prognosis—addressing 23 critical clinical questions and offering practical, evidence-based recommendations to guide more standardized and consistent care.

In formulating this expert consensus, recommendations were graded based on the strength of available evidence, clinical consistency, and expert agreement. The grading system is adapted from internationally recognized frameworks such as the GRADE methodology (6), categorizing recommendations as strong (Grade 1) or conditional (Grade 2), and quality of evidence as high (A), moderate (B), or low (C). For example, the recommendation that chromosomal microarray analysis (CMA) be offered to all fetuses with ventriculomegaly—including isolated cases—is graded as 1A, based on multiple cohort studies and meta-analyses demonstrating a substantial diagnostic yield of submicroscopic pathogenic variants (7) (Table 1).

**Table 1:** Expert recommendation grades and evidence levels.

Recommendation grade	Strength of recommendation	Quality of evidence	Typical source
1A	Strong recommendation	High-quality evidence (e.g., RCTs or meta-analyses)	Systematic review of RCTs or strong RCTs
1B	Strong recommendation	Moderate-quality evidence (e.g., consistent observational studies)	Well-done cohort studies or limited RCTs
1C	Strong recommendation	Low-quality evidence (e.g., expert opinion or case series)	Expert consensus or clinical experience
2A	Weak recommendation	High-quality evidence (e.g., RCTs or meta-analyses)	Systematic review of RCTs or strong RCTs
2B	Weak recommendation	Moderate-quality evidence (e.g., consistent observational studies)	Well-done cohort studies or limited RCTs
2C	Weak recommendation	Low-quality evidence (e.g., expert opinion or case series)	Expert consensus or clinical experience

RCTs, randomized controlled trials.

Similarly, the guidance that delivery timing and mode should follow standard obstetric indications in isolated mild ventriculomegaly is graded as 1C, reflecting consistent observational evidence and expert consensus in the absence of randomized controlled trials (8, 9). Where evidence remains limited—such as in the long-term neuropsychological surveillance of infants with isolated mild ventriculomegaly—recommendations are made conditionally (Grade 2B), emphasizing individualized decision-making informed by emerging data and multidisciplinary judgment.

## Material and methods

This consensus was approved by the Ethics Committee of West China Second University Hospital, Sichuan University (Approval No. 2024320) and registered with the Chinese Clinical Trial Registry (ChiCTR2400093980). To ensure scientific rigor and broad clinical applicability, this expert consensus was developed through a structured Delphi methodology. The process began with a systematic literature review and preliminary consultation with selected domain experts via in-person interviews and online discussions. Based on these inputs, the research team drafted the first-round expert questionnaire. This was followed by a multidisciplinary consultation involving specialists from obstetrics, neuroimaging, genetics, pediatric neurology, rehabilitation, nursing, and health informatics, who provided comprehensive feedback to refine the content. The revised version formed the basis for the second-round survey.

We conducted an literature search covering publications from 2000 to 2025, including high-quality evidence such as books, clinical trials, guidelines, meta-analyses, multicenter studies, randomized controlled trials, reviews, and systematic reviews related to fetal ventriculomegaly. A total of 42 key references were identified and incorporated into the revised manuscript, with their quality assessed and summarized in Supplementary Table S1.

A panel of 20–30 experts, each with over 10 years of professional experience in relevant fields, participated in the second-round Delphi survey. Their responses were analyzed, and further modifications were made, particularly addressing areas of persistent disagreement. This led to the development of a third-round questionnaire, which was redistributed to the expert panel—approximately half of whom had participated in the previous round—to collect final feedback and assess the level of consensus reached.

The exact number of participating experts from each specialty (obstetrics *n* = 8, fetal imaging *n* = 5, medical genetics *n* = 4, neonatology *n* = 3, neurology *n* = 3, rehabilitation *n* = 2, nursing *n* = 2, and informatics *n* = 2), the predefined consensus threshold (≥75% agreement), and the handling of disagreements through targeted discussions followed by re-voting in subsequent rounds. Attrition rates across the three Delphi rounds were also reported (Round 1: 29/29 responses; Round 2: 27/29 responses; Round 3: 25/29 responses), and reasons for non-response were documented. In addition, the unresolved disagreements after three rounds were addressed through in-person expert panel meetings to reach final consensus. Following statistical analysis of the responses, a draft version of the consensus was compiled. This draft underwent rigorous review through in-person expert panel meetings and cross-disciplinary discussions. The final version was established after thorough deliberation, ensuring that the resulting recommendations are scientifically sound, evidence-informed, and clinically relevant.

## Results

### Questions and recommendations

#### Advances in the precision diagnosis and etiological Spectrum of fetal ventriculomegaly

1. **Should diagnostic thresholds for fetal ventriculomegaly be adjusted based on gestational age, sex, or head circumference?**

Fetal ventriculomegaly is traditionally defined on sonographic scan as an atrial diameter (AD) ≥ 10 mm measured in the axial transventricular plane during the mid-trimester examination, regardless of gestational age, fetal sex, or head circumference; this threshold is also applied for diagnoses made in the third trimester (2). Studies show that normal AD remains relatively stable (≈4.5–7.6 mm) between 15 and 40 weeks GA (10). Current consensus, including SMFM guidelines, does not adjust thresholds based on sex or head size, although individual head circumference may be relevant when macrocephaly coexists (11). However, emerging evidence suggests that sex-based differences may exist: some cohorts report higher detection of genetic variants in male fetuses with ventriculomegaly (≈19.1 % vs. 5.5% in females) (12). Despite these observations, there is no robust evidence justifying sex- or biometry-adjusted AD thresholds at present.

**Recommendation:** Maintain standard threshold (AD ≥10 mm) regardless of GA, sex, or head size

**Grade: 1B**—Based on consistent observational data and current guidelines; no robust RCT or high-level evidence supports modifying thresholds.

2. **How should fetuses in the “grey zone” of 12–13 mm be stratified and managed?**

The categorization of ventriculomegaly is based on sonographic measurement of the atrial diameter (AD) at the level of the lateral ventricles on a standard axial transventricular plane. Ventriculomegaly is typically classified as mild (10–12 mm), moderate (13–15 mm), and severe (≥15 mm) to enhance prognostic clarity (7, 13). Fetuses in the 12–13 mm “grey zone” warrant more precise stratification due to variable outcomes. Most isolated mild cases (10–12 mm) have a favorable prognosis (>90% normal neurodevelopment), while moderate cases (≥13 mm) show higher risk (∼10%–40%) (11). For grey-zone cases, recommendations include serial neurosonographic monitoring, fetal MRI, and offering invasive genetic testing such as CMA regardless of isolation status (14, 15). A flowchart including dynamic tracking of AD progression, side differences, and imaging/genetic adjuncts is sensible in expert consensus frameworks.

**Recommendation:** It is suggested that fetuses with an atrial diameter of 12.1–12.9 mm be managed as moderate ventriculomegaly, with individualized care including serial ultrasound, fetal MRI, and CMA testing.

**Grade: 1C**—Supported by cohort studies and expert consensus; benefits of additional imaging and genetic testing outweigh risks.

3. **Should asymmetric or evolving ventriculomegaly be incorporated into diagnostic criteria?**

Yes. While classic definitions focus on maximal atrial width, both lateral asymmetry and dynamic longitudinal change should be considered. Bilateral ventriculomegaly is associated with a higher yield of pathogenic variants than unilateral cases (∼16.5 % vs. ∼8.6 %) (12, 14). Progressive dilation during pregnancy occurs in ∼13–16 % of moderate cases and is associated with worse neurodevelopmental outcomes, supporting dynamic follow-up protocols (13). As such, diagnostic frameworks should incorporate both side-to-side differences and temporal trends to refine risk assessment.

**Recommendation:** Diagnostic criteria should include lateral asymmetry and dynamic progression

**Grade: 1B**—Strong biological plausibility and cohort evidence show their association with adverse outcomes and increased diagnostic yield.

4. **Are non-traditional maternal factors—such as immune or metabolic dysfunction—underestimated in etiology?**

Most guidelines emphasize structural anomalies, infection (e.g., CMV, toxoplasma), and genetic causes. However, emerging literature suggests that maternal immune factors (e.g., antiphospholipid antibodies, ANA) and metabolic derangements might contribute to ventriculomegaly though high-quality RCT data are lacking (16). Currently no RCTs or meta-analyses directly address these associations. Nonetheless, case-series data and mechanistic hypotheses suggest a need for future research into maternal systemic or inflammatory influences as underexplored risk domains. Expert consensus should highlight this gap and recommend targeted research and screening protocols.

**Recommendation:** Currently insufficient evidence to support routine screening

**Grade: 2C**—Limited evidence base, primarily case series and mechanistic hypotheses; recommendation is conditional and highlights a research gap.

5. **What is the role of genomics [CMA/whole exome sequencing (WES)] and multi-omics in etiological evaluation?**

CMA significantly improves detection of pathogenic copy number variants (CNVs): incremental yield over karyotype in ventriculomegaly is ∼8–12%—even in mild isolated cases—and totals detection rates of ∼9–16% overall (12, 17). A meta-analysis of prenatal exome sequencing (ES) in structurally abnormal neonates found an additional diagnostic yield of approximately 31 % when compared with normal karyotype and CMA results; yield is higher (≈45%) in non-isolated severe cases (18, 19). Although most data concern severe structural anomalies, similar incremental benefits likely apply in moderate ventriculomegaly, especially when MRI reveals additional anomalies or when ventricular dilation is bilateral. As such, integrating CMA and ES (or targeted panels) into the diagnostic pathway supports higher diagnostic precision and better-informed prenatal counseling (20).

**Recommendation:** CMA should be offered for all cases of ventriculomegaly; WES/ES considered in moderate/severe cases

**Grade: 1A**—High-level evidence from meta-analyses supports significant diagnostic yield; guideline-backed recommendation.

### Summary of this section

This expert consensus supports the continued use of a fixed threshold (AD ≥10 mm) for diagnosing fetal ventriculomegaly across gestational ages and biometric profiles (Grade 1B). For fetuses within the 12–13 mm “grey zone,”. It is suggested that fetuses with an atrial diameter of 12.1–12.9 mm be managed as moderate ventriculomegaly. Individualized evaluation through serial neurosonography, MRI, and chromosomal microarray is recommended to refine prognostic stratification (Grade 1C). Asymmetric or progressively enlarging ventriculomegaly should be recognized as clinically meaningful and incorporated into diagnostic frameworks (Grade 1B). While traditional etiologies remain predominant, emerging hypotheses implicating maternal immune-metabolic dysfunction merit further research, though not yet routine clinical application (Grade 2C). Genomic technologies—particularly CMA and exome sequencing—have proven value in uncovering underlying etiologies and should be integrated into the diagnostic algorithm for all cases, with WES prioritized for non-isolated or moderate/severe presentations (Grade 1A).

#### Systematic evaluation of mild to moderate fetal ventriculomegaly

6. **How can prenatal ultrasound and MRI be combined into a standardized workflow to maximize detection of anomalies?**

The ENSO Working Group meta-analysis by Di Mascio et al. demonstrated that in fetuses with mild to moderate ventriculomegaly (VM, 10–15 mm), prenatal MRI identified additional CNS anomalies in approximately 5%–16% of cases that were missed on dedicated neurosonography, and altered perinatal management in 3%–5% (21). A structured pathway is recommended: Begin with comprehensive neurosonography using standardized axial, coronal, and sagittal views. If VM ≥10 mm is detected, proceed to fetal MRI regardless of isolation status. MRI should ideally be scheduled after detailed ultrasound, with preference for third trimester timing when clinically feasible (see Q3) (22). Multidisciplinary review (perinatology, neuroradiology, genetics) ensures optimal interpretation and counseling (11, 23, 24).

**Recommendation:** Implement a structured protocol of neurosonography followed by fetal MRI for VM ≥10 mm, regardless of isolation status, with multidisciplinary review.

**Grade: 1A**—Supported by meta-analyses and international guidelines; strong evidence for improved anomaly detection and clinical decision-making impact.

7. **What advantages does fetal MRI offer in detecting occult central nervous system anomalies?**

Fetal MRI has superior contrast resolution, is less affected by maternal habitus or oligohydramnios, and can more reliably identify anomalies such as agenesis or hypoplasia of the corpus callosum (ACC), cortical malformations, posterior fossa lesions, and white-matter abnormalities (22, 25). ENSO data indicate that associated anomalies—particularly cortical and white matter disorders—are more frequently detected by MRI than by ultrasound alone in VM cases during the third trimester (13, 26). For ACC specifically, MRI detected additional structural anomalies in about 11% when ultrasound suggested isolated ACC (27, 28).

**Recommendation:** Use fetal MRI to identify structural anomalies during the third trimester, especially corpus callosum abnormalities and cortical malformations, that may be missed on ultrasound.

**Grade: 1A**—Based on multiple high-quality cohort studies and meta-analyses demonstrating superior diagnostic yield compared to ultrasound alone.

8. **Should MRI timing (mid- vs. late gestation) be individualized?**

Yes. The meta-analysis by Di Mascio et al. found no significant difference in detection rates of additional anomalies before or after 24 weeks (*p* = 0.265), but third-trimester MRI may improve detection of cortical or hemorrhagic lesions (21). Thus, MRI timing should be tailored: Early MRI (mid-trimester) is useful for rapid evaluation after ultrasound. Late MRI (third trimester) may offer better visualization of cortical development, white matter maturation, or hemorrhage. Selection of MRI timing should consider suspected lesion type, fetal gestational age, and availability (29).

**Recommendation:** Tailor MRI timing based on clinical context, lesion suspicion, and gestational age; late MRI may be optimal for cortical and white matter evaluation.

**Grade: 1B**—Moderate evidence from meta-analysis and expert consensus supports individualized scheduling, though no RCTs exist.

9. **If ventriculomegaly appears isolated, should invasive genetic testing (CMA or targeted panels) be routinely recommended?**

Yes. International consensus, including the review by Giorgione et al., recommends offering chromosomal microarray analysis (CMA) to all fetuses with VM, even if isolated on imaging, due to elevated risk of submicroscopic CNVs (11, 21). While direct RCTs are not available, meta-analyses show that non-trivial proportions of apparently isolated VM harbor genetic aberrations detectable only by CMA or exome sequencing (17, 20). In addition, in line with the 2018 AJOG expert consensus on fetal ventriculomegaly, concurrent testing for congenital infections—particularly CMV and toxoplasmosis (TOX), and Zika virus in endemic regions—should also be considered as part of the diagnostic work-up to ensure comprehensive etiological evaluation (7). Hence, invasive genetic testing should be offered systematically.

**Recommendation:** Offer CMA to all fetuses with VM, including isolated cases; consider WES for unresolved or non-isolated cases.

**Grade: 1A**—Strong meta-analytic evidence for non-negligible CNV detection in isolated VM; widely supported by expert consensus and practice guidelines.

10. **How should assessment be adapted in multiple gestations or when soft markers (e.g., short nasal bone, echogenic bowel) co-occur?**

In such complex scenarios, enhanced surveillance is warranted, including neurosonography and MRI when feasible. For isolated, non-progressive mild VM, the association with single nucleotide variants (SNVs) appears weak, and the presence of soft markers alone should not be overinterpreted as indicating increased structural risk. Genetic testing, particularly CMA, may be considered based on the overall clinical context, while exome sequencing should be reserved for cases with additional risk factors or unresolved findings. Individualized evaluation pathways should incorporate multidisciplinary input and precise imaging protocols.

**Recommendation:** Enhanced surveillance and CMA may be considered based on clinical context, while WES should be reserved for cases with additional risk factors or unresolved findings.

**Grade: 2C**—Evidence is low, and current practice is heterogeneous; represents an important research priority rather than a clinical directive.

11. **Are there defined criteria for dynamic monitoring of VM during late pregnancy? How are “improvement” or “worsening” delineated?**

Although universally accepted standards are lacking, clinical series and meta-analyses identify dynamic trends as prognostic indicators: Progression of atrial diameter (AD) ≥2 mm over serial scans is associated with worse neurodevelopmental outcome (16). Worsening from mild (<12 mm) to moderate range (>13 mm), or onset of bilateral involvement, indicates elevated risk (29). Improvement (decrease in AD), stability, vs. progression should be defined in serial monitoring protocols, ideally spaced 2–4 weeks apart in mid to late pregnancy.

**Recommendation:** Use ≥2 mm change in AD, progression from unilateral to bilateral, or shift from mild to moderate as indicators of worsening; perform serial scans every 2–4 weeks.

**Grade: 2B**—Based on observational data and expert opinion; no universal standard, but growing agreement on prognostic value of dynamic trends.

12. **Is there any validated scoring system to predict adverse outcomes in fetuses with mild-moderate ventriculomegaly?**

Currently, no widely validated scoring system exists. However: Prognostic factors identified in systematic reviews include maximal AD, bilateral vs. unilateral dilation, progression over time, and coexistence of structural or genetic anomalies (13, 30).

Many centers utilize composite risk assessment combining imaging (ultrasound/MRI) and genetic findings, but formal predictive models remain under development.

Expert consensus should recommend that future research establishes prognostic scoring tools based on large cohort data.

**Recommendation:** No validated model exists; future research should focus on establishing predictive scoring systems incorporating imaging and genetic data.

**Grade: 2C—**Evidence is low, and current practice is heterogeneous; represents an important research priority rather than a clinical directive.

### Summary of this section

A stepwise imaging strategy combining detailed neurosonography with fetal MRI is strongly recommended for fetuses with ventriculomegaly, as it significantly improves the detection of occult CNS anomalies (Grade 1A). MRI offers particular advantages in identifying abnormalities such as agenesis of the corpus callosum and cortical dysplasia (Grade 1A), and its timing should be individualized based on the suspected lesion and gestational age (Grade 1B). Genetic evaluation using chromosomal microarray is advised even in isolated cases of VM due to the substantial risk of pathogenic CNVs (Grade 1A), and the presence of soft markers warrants enhanced genetic and imaging assessment (Grade 2C). Although there are no universally accepted criteria, dynamic changes in ventricular width or laterality can help guide clinical management and should be monitored serially (Grade 2B). At present, no validated scoring system exists for predicting neurodevelopmental outcomes, highlighting the need for future prospective studies to develop evidence-based prognostic models (Grade 2C).

#### Antenatal management strategies for fetal ventriculomegaly

13. **Does mild to VM necessitate modification of antenatal surveillance, such as increased fetal movement monitoring or intrauterine assessment?**

According to the SMFM (Society for Maternal-Fetal Medicine) 2018 guidance, mild (10–12 mm) to moderate (13–15 mm) VM should trigger serial ultrasound follow-up to evaluate progression, but routine biophysical profile or non-stress testing is not generally indicated unless placental insufficiency, fetal growth restriction, or amniotic fluid abnormalities coexist (GRADE 1C) (7, 8, 31). The WHO ROMANCE meta-analysis also emphasises that VM alone does not predict fetal compromise requiring altered fetal movement surveillance. However, in fetuses with progressive dilation (defined as ≥2 mm increase over serial scans), increased surveillance—including more frequent growth scans and Doppler or biophysical assessment—is reasonable (4, 5).

**Recommendation:** For isolated mild to moderate VM, routine antenatal surveillance (e.g., fetal movement counts or biophysical profiles) does not require escalation unless coexisting complications are present.

**Grade: 1C**—Based on expert guidelines and consistent observational data, but limited by the absence of RCTs or high-level comparative studies.

14. **Should pregnancies with VM plus other soft markers—but no confirmed structural abnormality—be managed under an “enhanced surveillance” pathway rather than routine care?**

Yes. Pregnancies combining VM with additional soft markers—particularly increased nuchal fold thickness—are associated with a higher risk of chromosomal or genetic anomalies, even when no definite structural malformations are identified. SMFM and other expert consensus advise offering chromosomal microarray and detailed neurosonography as part of an enhanced evaluation in such cases (7). Furthermore, serial ultrasound follow-up every 4 weeks is suggested to monitor the evolution of the ventriculomegaly, even if it initially appears isolated. This level of follow-up exceeds routine prenatal surveillance for low-risk pregnancies.

**Recommendation:** Yes. In the presence of additional soft markers, enhanced prenatal surveillance and genetic testing (e.g., CMA) should be offered, even without confirmed structural anomalies.

**Grade: 1B**—Supported by large cohort data and consensus guidelines; presence of soft markers is a recognized risk factor for chromosomal abnormalities.

15. **How should management and decision-making proceed during pregnancy when ventriculomegaly shows progressive enlargement?**

Fetuses whose ventricular width increases by ≥2 mm or transitions from mild to moderate range are classified as progressive VM, which is associated with higher neurodevelopmental risk (up to ∼22% adverse outcomes) (5). In these cases, recommendations include: Intensified ultrasound monitoring, repeated every 2–4 weeks to assess ventricular trends, fetal biometry, amniotic fluid volume, and potential abnormalities in other organ systems. Prompt fetal MRI (if not already performed) to detect occult structural CNS anomalies. Invasive prenatal diagnosis, including CMA with sequential or combined whole exome sequencing (WES), to refine etiological evaluation and guide counseling. Multidisciplinary consultation (maternal–fetal medicine, genetics, neonatology, neurosurgery) for counseling. Discussing potential perinatal planning including referral to tertiary center, neonatal imaging at birth, and early neurodevelopmental follow-up (29). This approach aligns with high-quality observational data and expert consensus that highlight the prognostic importance of dynamic changes rather than initial ventricular width alone.

**Recommendation:** Progressive VM (≥2 mm increase or worsening classification) should prompt intensified ultrasound surveillance, fetal MRI, and multidisciplinary consultation for perinatal planning.

**Grade: 1B**—High-quality cohort studies support the prognostic value of progression; expert consensus consistently endorses intensified follow-up in these cases.

### Summary of this section

In pregnancies with isolated mild to moderate ventriculomegaly, routine antenatal surveillance need not be intensified unless additional obstetric complications arise, such as fetal growth restriction or amniotic fluid abnormalities (Grade 1C). However, if soft markers—such as echogenic bowel or nasal bone hypoplasia—are also present, even without definitive structural malformations, these cases should be managed under an enhanced surveillance framework, including serial imaging and chromosomal microarray testing (Grade 1B). Furthermore, when ventriculomegaly progresses during gestation—defined as a ≥2 mm increase in atrial diameter or transition in severity—this dynamic change is associated with increased neurodevelopmental risk and warrants more intensive management. This includes repeated imaging every 2–4 weeks, prompt fetal MRI, and multidisciplinary counseling to guide delivery planning and postnatal follow-up. Invasive prenatal diagnosis, including CMA with sequential or combined whole exome sequencing (WES), to refine etiological evaluation and guide counseling. (Grade 1B).

#### Timing and mode of delivery in fetuses with ventriculomegaly

16. **Can isolated mild ventriculomegaly follow routine term vaginal delivery?**

Yes. The Society for Maternal-Fetal Medicine (SMFM) 2018 Expert Consult Series strongly recommends that both timing and mode of delivery for isolated mild (10–12 mm) or moderate (13–15 mm) ventriculomegaly be based on standard obstetric indications. There is no evidence from randomized trials that cesarean section or early delivery improves neonatal or neurodevelopmental outcomes in these cases (GRADE 1C) (7). Meta-analytic data show that most infants with isolated mild VM (>90%) experience normal neurodevelopment and require no deviation from routine delivery pathways.

**Recommendation:** Yes. Delivery timing and mode should follow standard obstetric indications for isolated mild VM.

**Grade: 1C**—Based on consistent observational studies and expert consensus; no randomized trial evidence available.

17. **Should a more proactive delivery strategy be adopted for moderate VM with mild growth restriction or amniotic fluid abnormalities?**

Current guidelines and observational studies suggest no definitive benefit from elective early delivery or cesarean section purely due to ventriculomegaly, even in the presence of mild fetal growth restriction or oligohydramnios. Management should remain individualized based on obstetric risk factors. For moderate VM coexisting with significant obstetrical complications, more active fetal surveillance and timely delivery when standard clinical thresholds are met (e.g., nonreassuring fetal monitoring, worsening growth restriction) is prudent, but routine early delivery is not indicated without other clinical indications (7).

**Recommendation:** No. Delivery decisions should remain individualized and based on standard obstetric risk indicators, not solely on the presence of ventriculomegaly.

**Grade: 2C—**Supported by observational data and guidelines; lacks high-level comparative trials.

18. **Is neonatal brain imaging immediately after birth recommended, and who should lead this?**

Yes. Postnatal cranial imaging is advised when prenatal VM has been detected, especially in moderate cases or if additional risk factors are present. Cranial ultrasound (CrUS) is the typical first-line modality in newborns, often performed by neonatologists or trained pediatric radiologists within the first few days of life to exclude unseen complications such as hemorrhage or persistent ventriculomegaly (32, 33). If abnormalities persist or more detailed assessment is needed, a neonatal brain MRI at term-corrected age may be considered, ideally under the guidance of pediatric neurology and neuroradiology teams (34). Ownership of the imaging and interpretation should rest with the pediatric/neonatal team, in consultation with neurology or neurosurgery as appropriate.

**Recommendation:** Yes. Postnatal cranial ultrasound should be performed in neonates with prenatal VM, especially moderate or non-isolated cases. MRI may follow based on clinical findings. Pediatric radiology or neurology should lead the imaging evaluation.

**Grade: 1B**—Moderate evidence from large cohorts and clinical practice guidelines supports its diagnostic value and clinical utility.

19. **Do different modes of delivery have any microstructural impact on neonatal brain structures?**

There is no high-quality evidence—such as RCTs or meta-analyses—demonstrating that vaginal vs. cesarean delivery materially affects neonatal brain structures or long-term neurological outcomes in fetuses with ventriculomegaly (34). The literature consistently supports that delivery mode should reflect obstetric indications rather than perceived neuroprotective advantage. Macrocephaly is rare, and cesarean delivery might be considered only in exceptional situations such as markedly enlarged head circumference (e.g., >40 cm), where standard obstetric risks dictate operative delivery (2).

**Recommendation:** No. There is no evidence supporting the use of cesarean section over vaginal delivery for neuroprotection in VM. Mode of delivery should follow obstetric indications.

**Grade: 1C**—No RCTs or meta-analyses available; consistent observational evidence and expert consensus support this approach.

### Summary of this section

In cases of isolated mild ventriculomegaly, term vaginal delivery following standard obstetric criteria is appropriate and does not require alteration based on fetal brain findings (Grade 1C). Even in moderate ventriculomegaly coexisting with mild obstetric complications (e.g., borderline growth restriction or amniotic fluid anomalies), a proactive delivery approach is not routinely justified, and decisions should be guided by conventional obstetric thresholds (Grade 2C). Postnatal cranial imaging is recommended for all moderate or complex VM cases, with cranial ultrasound as the first-line modality and MRI considered for further evaluation. These should be managed under pediatric or neonatal leadership, in consultation with neurology as needed (Grade 1B). Finally, there is no scientific evidence indicating that delivery mode influences neonatal brain microstructure or long-term neurodevelopment in ventriculomegaly cases; cesarean section should be reserved for obstetric indications rather than presumed neuroprotection (Grade 1C).

#### Short- and long-term outcomes of mild to moderate fetal ventriculomegaly

20. **Can prognostic scoring models based on prenatal variables (maximal atrial width, progression, co-existing anomalies) accurately predict outcome?**

While no universally validated prognostic scoring system currently exists, multiple studies have identified consistent prenatal risk factors. A meta-analysis of 652 isolated mild ventriculomegaly (≤15 mm) cases found neurodevelopmental delay in 7.9% (95% CI: 4.7–11.1%), with false-negative postnatal imaging in ∼7.4% (9, 35). Additionally, a large cohort study of 324 cases demonstrated that early diagnosis (≤24 + 6 weeks), non-isolated ventriculomegaly, and intrauterine progression significantly increased risk (OR: 2.86, 2.62, and 11.15 respectively) (19, 35). Thus, combining variables such as initial gestational age at diagnosis, laterality, evolution, and structural context into a composite risk model may provide meaningful prognostic stratification, though formal scoring systems remain in development.

**Recommendation:** Composite prenatal risk models are promising but not yet formally validated; their development is encouraged for clinical use.

**Grade: 2C**—Supported by consistent cohort and meta-analytic evidence, but formal scoring tools are not yet widely implemented or prospectively validated.

21. **What domains of neurodevelopmental risk are most commonly affected?**

Systematic reviews show that even isolated mild ventriculomegaly carries an elevated risk (∼8%–11%) of neurodevelopmental impairments—most commonly affecting language, fine and gross motor skills, and mild global developmental delay (11, 35, 36). Severe VM is associated with a relative risk of 4.24 for neurodevelopmental delay compared to mild cases, and nearly 45% of severe cases exhibit developmental delay (15, 32).

**Recommendation:** Language, motor, and cognitive domains are most at risk, and should be a focus of postnatal developmental surveillance.

**Grade: 1B—**Based on multiple systematic reviews and large observational cohorts indicating reproducible patterns of developmental vulnerability.

22. **Should high-risk postnatal follow-up protocols be recommended, and how early should interventions begin?**

Yes. The literature supports structured, risk-stratified follow-up. For fetuses with VM plus risk factors—especially early onset, progression, or co-existing anomalies—coordination with pediatric neurology and developmental pediatrics soon after birth is recommended. Initial neurodevelopmental screening should commence by 6 months of age, with ongoing formal testing (Bayley, ASQ-3) through early childhood, particularly in those with identified prenatal risk (33). Early intervention—when developmental delays are identified—can improve function in language, motor coordination, and cognitive domains (8).

**Recommendation:** Yes. Structured follow-up beginning by 6 months of age is advised for infants with prenatal risk factors (e.g., progression, early onset, non-isolation).

**Grade: 1B**—Strong support from longitudinal studies and expert consensus demonstrating benefit of early developmental screening and intervention.

23. **If a child is born with isolated mild ventriculomegaly and normal neurological examination, is long-term neuropsychological follow-up still warranted?**

Yes, especially in cases with early-onset or progression prenatally, despite normal newborn examinations (35). While most isolated mild VM cases (≈ 90%) have normal outcomes, subtle vulnerabilities—such as attention, language, or social cognition deficits—may emerge later (37, 38). Therefore, periodic developmental surveillance up to at least school age is advisable. Even where initial pediatric neurology assessment is normal, follow-up through preschool years ensures early detection of subtle behavioral or learning difficulties and enables timely intervention (7, 39).

**Recommendation:** Yes. Even with normal neonatal findings, long-term developmental surveillance is recommended due to risk of subtle late-emerging deficits.

**Grade: 2B—**Evidence suggests a minority of such children may develop learning or behavioral issues later; recommendation is based on best practices and preventive value.

### Summary of this section

Although a universally validated prognostic scoring system for fetal ventriculomegaly is not yet available, current evidence supports stratifying risk based on prenatal variables such as atrial width, gestational timing, lesion laterality, and progression over time (Grade 2C). Neurodevelopmental impairments, particularly in language, fine and gross motor skills, and global functioning, are among the most frequently reported outcomes in affected children (Grade 1B). High-risk neonates—especially those with early-onset, progressing, or non-isolated ventriculomegaly—should undergo structured follow-up beginning by six months, including formal developmental testing and early intervention if delays are identified (Grade 1B). Even infants with isolated mild VM and reassuring newborn exams benefit from long-term neuropsychological monitoring, as subtle deficits may not manifest until preschool or school age (Grade 2B).

## Discussion

This expert consensus comprehensively addresses the clinical challenges and knowledge gaps surrounding fetal ventriculomegaly, one of the most frequently encountered prenatal CNS anomalies. By organizing 23 key questions across five domains—diagnosis, evaluation, antenatal management, delivery planning, and prognosis—it offers a structured and evidence-based framework to support clinical decision-making. The summary of recommendations in this consensus is presented in Table 2. The consensus emphasizes precision in diagnostic criteria, advocates for standardized use of fetal MRI and genetic testing, and provides recommendations for individualized management strategies based on the severity and progression of ventriculomegaly.

**Table 2:** Summary of expert recommendations and grades.

No.	Clinical question	Recommendation summary	Strength of recommendation	Quality of evidence
1	Should diagnostic thresholds be adjusted by gestational age, sex, or head circumference?	Use fixed ≥10 mm threshold; no adjustment by GA, sex, or biometry	1B	Moderate
2	How to manage fetuses in 12–13 mm “grey zone”?	Stratify with MRI, serial ultrasound, and genetic testing (CMA)	1C	Low
3	Should asymmetric/dynamic VM be incorporated into diagnosis?	Yes, monitor bilateral involvement and progressive dilation	1B	Moderate
4	Are maternal immune/metabolic factors underestimated in VM etiology?	Currently insufficient evidence	2C	Low
5	Should CMA/WES be used in etiologic workup?	CMA for all cases; WES for unresolved or complex cases	1A	High
6	How to structure combined ultrasound–MRI workflow?	Begin with neurosonography → MRI → multidisciplinary review	1A	High
7	What are MRI's advantages in CNS anomaly detection?	Superior for ACC, cortex, posterior fossa, white matter	1A	High
8	Should MRI timing be individualized (mid vs. late gestation)?	Yes, based on anomaly type and GA	1B	Moderate
9	Should CMA be offered in isolated VM?	Yes, due to non-trivial CNV detection rate	1A	High
10	How to assess VM in twin pregnancy or with soft markers?	Enhanced evaluation with MRI, genetic testing, and expert review	2C	Moderate
11	Are there standard criteria for serial monitoring?	Progression = AD increase ≥2 mm or bilateral involvement; reassess every 2–4 weeks	2B	Moderate
12	Are prognostic scores validated for predicting outcome?	Not yet; risk stratification recommended based on current evidence	2C	Low
13	Does mild/moderate VM need enhanced fetal surveillance (e.g., BPP, NST)?	Not routinely, unless complications exist	1C	Low
14	Should soft markers without structural anomalies lead to enhanced pathway?	Yes, due to elevated risk	1B	Moderate
15	How to manage progressing VM during pregnancy?	Serial scans + MRI + multidisciplinary consultation + perinatal planning	1B	Moderate
16	Can isolated mild VM follow routine term vaginal delivery?	Yes, unless obstetric indications arise	1C	Low
17	Should delivery strategy change for moderate VM with FGR or Oligo?	No. Delivery decisions should base on standard obstetric risk indicators	2C	Low
18	Should newborns undergo cranial imaging post-delivery?	Yes, CrUS followed by MRI if necessary; led by neonatology/neuro teams	1B	Moderate
19	Does mode of delivery affect neonatal brain structure?	No evidence supporting neuroprotective benefit from cesarean	1C	Low
20	Can prognostic scoring models based on prenatal variables accurately predict outcome?	Composite prenatal risk models are promising but not yet formally validated.	2C	Low
21	What neurodevelopmental domains are most affected?	Language, motor, cognition most common	1B	Moderate
22	Should high-risk neonates have structured follow-up from birth?	Yes, start by 6 months, continue through early childhood	1B	Moderate
23	Is long-term surveillance needed after normal neonatal exam in isolated mild VM?	Yes, to detect subtle deficits emerging later (e.g., attention, learning)	2B	Moderate

RCTs, randomized controlled trials, GA, gestational age; CMA, chromosomal microarray analysis; WES, whole exome sequencing; VM, ventriculomegaly; AD, atrial diameter; CNS, central nervous system; ACC, agenesis of the corpus callosum; CNV, copy number variation; BPP, biophysical profile; NST, non-stress test; FGR: fetal growth restriction; Oligo, oligohydramnios, CrUS: cranial ultrasound.

A major strength of this consensus is its integration of current high-quality evidence, including cohort studies, meta-analyses, and international guidelines, with expert clinical judgment. Recommendations are graded using an adapted GRADE framework to reflect both the strength of the guidance and the underlying quality of evidence. In areas where definitive data remain limited—such as long-term neurodevelopmental surveillance or scoring model validation—conditional recommendations are made, encouraging further research and individualized care.

The recommendations regarding the application of CMA and WES are based on high-level evidence from meta-analyses and large cohort studies demonstrating their incremental diagnostic yield in fetuses with ventriculomegaly. Similarly, the prognostic role of ventricular progression and the advantages of MRI for detecting occult CNS anomalies are supported by consistent observational data and meta-analytic evidence, and are presented as strong recommendations (Grades 1A–1B). In contrast, statements regarding maternal immune and metabolic factors are now explicitly framed as preliminary research hypotheses, reflecting limited current evidence from case series and mechanistic studies, and are not included as formal clinical recommendations. Standardized definitions and universally accepted guidelines are currently lacking for several key prenatal issues, including the “grey zone” of 12–13 mm ventricular width, asymmetric or evolving ventriculomegaly, dynamic monitoring strategies in late pregnancy, and the management of progressive ventriculomegaly. In these areas, our recommendations were primarily based on expert consensus, whereby items supported by ≥85% agreement among the panel were assigned lower recommendation grades.

We recognize that access to advanced imaging modalities such as fetal MRI, and genetic technologies including CMA and WES, remains limited in many low- and middle-resource regions. Practical barriers—including cost-effectiveness considerations, availability of trained personnel, and infrastructure disparities—may influence the feasibility of implementing certain recommendations. Tailored strategies, such as regional referral networks, prioritized testing for higher-risk cases, and phased implementation, may help bridge these gaps. And this consensus was developed by a multidisciplinary panel based in China, and therefore reflects the healthcare infrastructure, clinical practice patterns, and resource availability of this context. While the core principles are aligned with international guidelines and evidence, adaptations may be needed to accommodate regional differences in healthcare systems, diagnostic resources, and population characteristics. We view this consensus as a framework that can be refined and validated through international collaboration and future multicenter studies.

Future research should focus on developing and validating robust prognostic models through large, multicenter prospective cohorts that integrate clinical, imaging, genetic, and maternal systemic factors. Advanced statistical methods and machine learning approaches can be leveraged to construct risk stratification tools with high predictive accuracy. International collaboration will be essential to ensure model generalizability and facilitate the establishment of standardized prognostic scoring systems for clinical use.

## Conclusion

This document aims to bridge the gap between evolving research and daily prenatal practice. By promoting standardized, multidisciplinary, and data-driven approaches to fetal ventriculomegaly, it strives to improve diagnostic accuracy, optimize perinatal outcomes, and facilitate longitudinal follow-up tailored to each fetus's risk profile. This consensus serves as both a clinical guide and a call for future studies to refine the management of this complex and heterogeneous condition.

## Data Availability

The raw data supporting the conclusions of this article will be made available by the authors, without undue reservation.
