# Supplementary material for: Expert consensus on fetal ventriculomegaly: evidence-based recommendations for 23 key clinical questions
Source: Front Pediatr. 2025 Oct 17;13:1678359. doi: 10.3389/fped.2025.1678359 (PMC12575118; doi:10.3389/fped.2025.1678359)
Supplement: Supplementary file 1 [file Table1.docx]

**Supplementary table 1: Evidence grading of key references for fetal ventriculomegaly consensus (2000–2025).**

| **No.** | **Reference** | **Study Type / Key Features** | **Evidence Grade** |
| --- | --- | --- | --- |
| 1 | Salomon 2011 | International guideline | 1B |
| 2 | Alluhaybi 2022 | Narrative literature review | 1C |
| 3 | Millischer 2019 | Retrospective cohort, small sample | 2C |
| 4 | Di Mascio 2024 | Systematic review / Meta-analysis (mild & moderate VM counseling) | 1A |
| 5 | Chen 2023 | Retrospective cohort study | 1B |
| 6 | Guyatt 2011 | GRADE methodology guideline | 1A |
| 7 | SMFM 2018 | Professional guideline | 1B |
| 8 | Wax 2003 | Narrative review | 1C |
| 9 | Wang 2024 | Retrospective cohort (324 cases) | 1B |
| 10 | Sapantzoglou 2025 | Systematic review | 1A |
| 11 | Giorgione 2022 | Clinical guidance / review | 1C |
| 12 | Yue 2024 | Retrospective cohort study | 1B |
| 13 | Zamlynski 2024 | Narrative review | 1C |
| 14 | Lok 2021 | Retrospective cohort (chromosomal abnormalities) | 1B |
| 15 | Gaglioti 2009 | Narrative review + retrospective data | 1C |
| 16 | D’Addario 2023 | Narrative review | 1C |
| 17 | Wadt 2012 | Case series (6q deletions) | 2C |
| 18 | Mustafa 2023 | Systematic review / Meta-analysis (WES in prenatal) | 1A |
| 19 | Wang 2018 | Focused review (CNV) | 1C |
| 20 | Sun 2021 | Meta-analysis (CMA vs karyotype) | 1A |
| 21 | Di Mascio 2019 | Systematic review / Meta-analysis (MRI) | 1A |
| 22 | Cardoen 2011 | Retrospective study + review | 1B |
| 23 | Hutter 2023 | Prospective observational study | 1B |
| 24 | Putbrese 2017 | Review / case discussion | 2C |
| 25 | Zimmerman 2005 | Review / case series | 1C |
| 26 | Yu 2024 | Technical review (MRI) | 1C |
| 27 | Vola 2023 | Retrospective observational study | 1B |
| 28 | Severino 2022 | Retrospective observational study | 1B |
| 29 | Mirsky 2020 | Review | 1C |
| 30 | Ali 2024 | Systematic review / Meta-analysis (prognosis) | 1A |
| 31 | Gomez-Arriaga 2023 | Prospective / mid-term outcome study | 1B |
| 32 | Guillot 2020 | Guideline-type review (neonatal imaging) | 1B |
| 33 | Wang KC 2011 | Review + case series (postnatal management) | 1C |
| 34 | SMFM 2020 | Professional guideline | 1B |
| 35 | Pagani 2014 | Systematic review / Meta-analysis (isolated mild VM outcomes) | 1A |
| 36 | Laskin 2005 | Systematic review (prognosis) | 1A |
| 37 | Melchiorre 2009 | Clinical guidance / review | 1C |
| 38 | Wyldes 2004 | Retrospective observational study | 1B |
| 39 | Scelsa 2018 | Single-center study + literature review | 1B |
